# Detection of gene mutation in the prognosis of a patient with arrhythmogenic right ventricular cardiomyopathy: a case report

**DOI:** 10.1186/s13256-023-04326-w

**Published:** 2024-02-10

**Authors:** Dinh Phong Phan, Tuan Viet Tran, Vo Kien Le, Tuan Viet Nguyen

**Affiliations:** 1 Cardiac Electrocardiogram and Electrophysiology Laboratory, Vietnam National Heart Institute, Bach Mai Hospital, 78 Giai Phong St., Hai Ba Trung, Hanoi, Vietnam; 2 Hanoi Medical University, 1 Ton That Tung St., Dong Da, Hanoi, Vietnam

**Keywords:** Arrhythmogenic right ventricular cardiomyopathy (ARVC), Gene muations, PKP2 gene, Case report

## Abstract

**Background:**

Arrhythmogenic right ventricular cardiomyopathy (ARVC), or more recently known as arrhythmogenic cardiomyopathy (ACM), is an heritable disorder of the myocardium characterized by progressive fibrofatty replacement the heart muscle and risk of ventricular arrhythmias and sudden cardiac death (SCD). We report a case study to demonstrate the role of gene mutation detection in risk stratification for primary prevention of SCD in a young patient diagnosed with ARVC.

**Case presentation:**

A 15-year-old Asian (Vietnamese) male patient with no history of documented tachyarrhythmia or syncope and a family history of potential SCD was admitted due to palpitations. Clinical findings and work-up including cardiac magnetic resonance imaging (MRI) were highly suggestive of ARVC. Gene sequencing was performed for SCD risk stratification, during which PKP2 gene mutation was found. Based on the individualized risk stratification, an ICD was implanted for primary prevention of SCD. At 6 months post ICD implantation, the device detected and successfully delivered an appropriate shock to terminate an episode of potentially fatal ventricular arrhythmia. ICD implantation was therefore proven to be appropriate in this patient.

**Conclusions:**

While gene mutations are known to be an important factor in the diagnosis of ARVC according to the 2010 Task Force Criteria and recent clinical guidelines, their role in risk stratification of SCD remains controversial. Our case demonstrated that when used with other clinical factors and family history, this information could be helpful in identifying appropriate indication for ICD implantation.

## Background

Arrhythmogenic right ventricular cardiomyopathy (ARVC), or more recently known as arrhythmogenic cardiomyopathy (ACM), is an heritable disorder of the myocardium characterized by progressive myocardial changes and risk of ventricular arrhythmias and sudden cardiac death (SCD). First described in the late 1970s and early 1980s [1], histopathological characterization of the right ventricle (RV) identifies multiple changes, most notably the presence of progressive fibrofatty or fat replacement of the myocardium, leading to RV dilatation and dysfunction [2]. This process forms heterogeneous zones of the myocardium correlated with arrhythmogenic substrates, which trigger the occurrence of ventricular tachycardias (VT) and ventricular fibrillation (VF) that can lead to SCD [3, 4]. However, recent studies have shown that biventricular involvement is more prevalent than previously thought and therefore, the term AVRC is being replaced by arrhythmogenic cardiomyopathy (ACM) [5] It has been reported that 73% of ARVC index patients carry mutations in genes encoding the desmosomal proteins [6, 7] necessary for the maintenance of stable intercellular connections. The prevalence of these gene mutations ranges from 28 to 58% [8, 9]. *TGFB3, RYR2, TTN, TNEM43, DES, DSP, PKP2, DSG2, DSC2, JUP, PLN, LMNA, SCN5A*, and *CTNNA3* have all been identified as playing a role in the pathogenesis of ARVC in recent studies [10]. This finding provides the foundation for the latest recommendation in European Society of Cardiology (ESC) guidelines as well as Heart Rhythm Society (HRS) consensus to consider gene mutations as a criterion for ARVC diagnosis and risk stratification [11, 12]. Nevertheless, the role of gene mutations in guiding treatment decisions remains controversial, particularly in the risk stratification of primary prevention of SCD [13]. We report a case study to demonstrate the role of gene mutation detection in risk stratification for treatment decisions.

## Case presentation

**Fig. 1:**
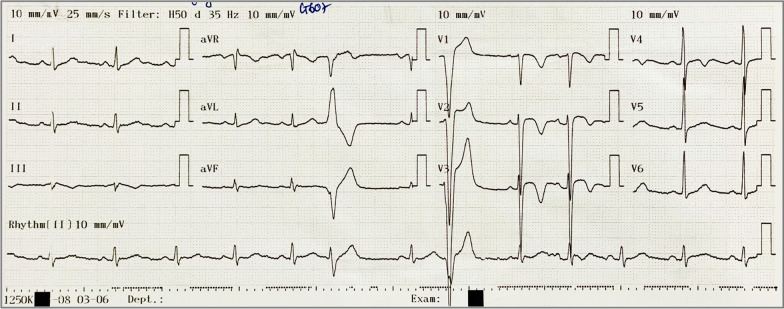
Electrocardiography on admission showing baseline rhythm was sinus rhythm, T wave inversion in leads V1–V3, with frequent and polymorphic premature ventricular complexes

A 15-year-old Asian (Vietnamese) male high school student as well as basketball player presented with the first-ever episode of palpitations. He had never had syncope or presyncope, documented arrhythmia or been diagnosed with cardiovascular diseases. His 27-year-old brother had died suddenly one year ago due to out of hospital cardiac arrest. Most likely cause of death was identified as arrhythmia by emergency physicians after other differentials such as cerebral vascular stroke, myocardial infarction, congenital and acquired cardiac disease were excluded by imaging, blood tests. On examination**,** his vital signs were normal with no clinical signs of heart failure. His electrocardiography (ECG) on admission (Fig. 1) showed frequent and various forms of premature ventricular complexes (PVC) with negative T waves in leads V1–V3.

**Fig. 2:**
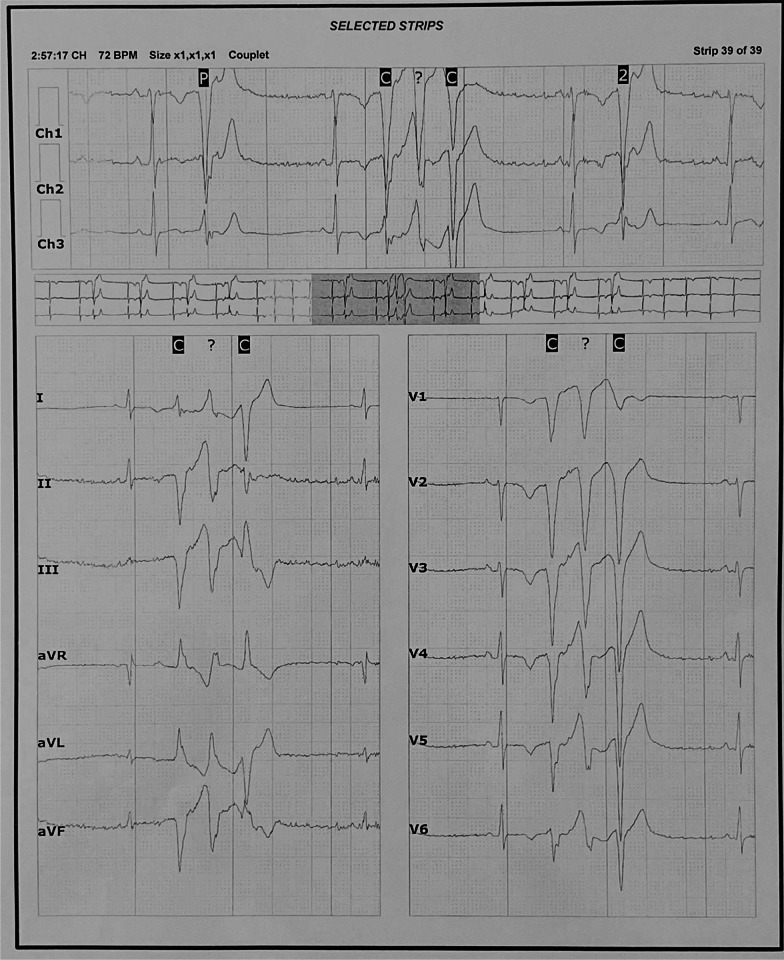
24-h ambulatory Electrocardiography: two consecutive premature ventricular complexes variants were recorded

Transthoracic echocardiography showed mildly dilated right ventricle with no other significant structural or functional abnormalities. The 24-h ECG ambulatory (Fig. 2) revealed significant PVC burden, accounting for 10.5% of the total number of heart beats. At least three distinct PVC morphologies were observed. There were occasionally coupled PVCs but no non-sustained or sustained VT was captured.

**Fig. 3:**
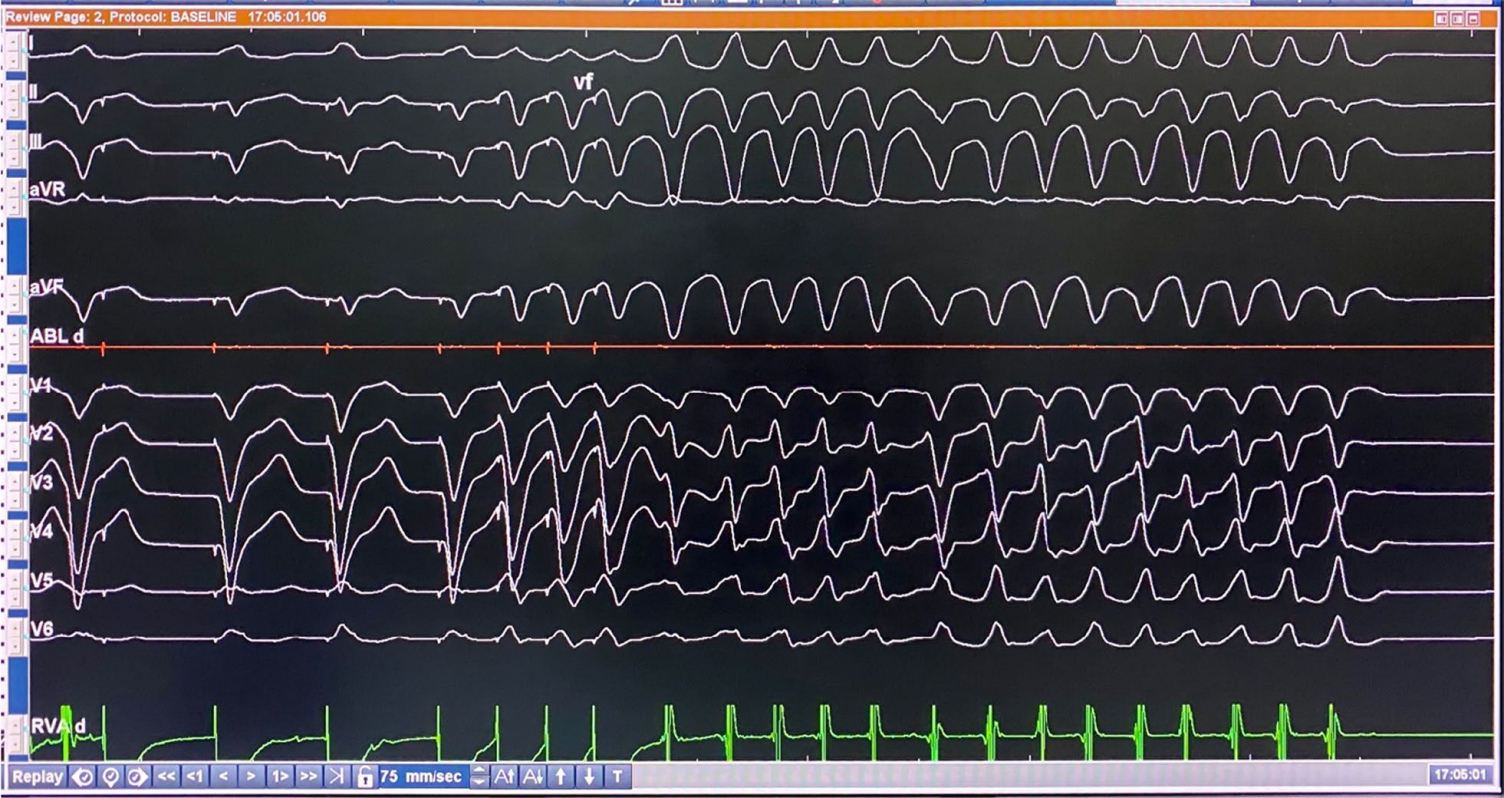
Programmed electrical stimulation induced a non-sustained ventricular tachycardia

Electrophysiology (EP) study was indicated for two purposes: (1) to ablate the frequent PVCs, and (2) to conduct programmed electrical stimulation (PES) to induce sustained ventricular arrhythmias for risk stratification. During the procedure, frequent PVCs with various patterns were observed. These PVCs originated from the RV at different sites of the postero-lateral wall, proximal to the tricuspid annulus. Multiple attempts of mapping and radiofrequency ablation failed to terminate all PVCs. PES delivered at the RV apex with two extra stimuli induced non-sustained episodes of VT repeatedly (Fig. 3).

**Fig. 4:**
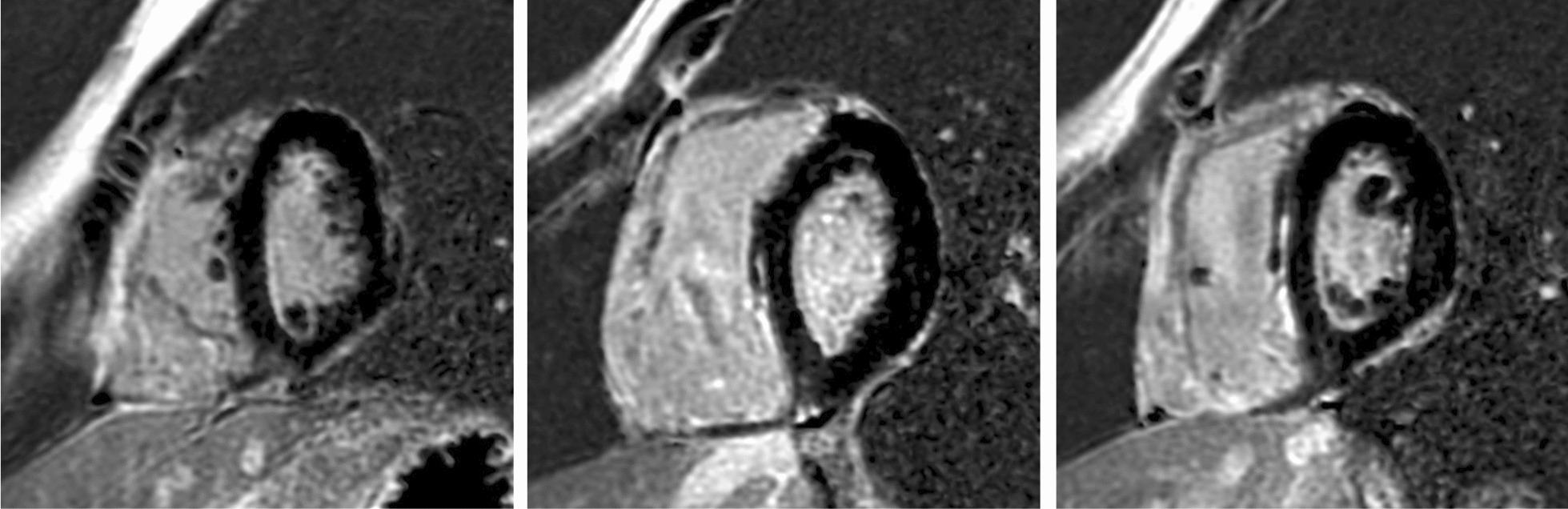
Cardiac Magnetic Resonance Imaging showed intramyocardial late gadolinium enhancement due to diffused fibrofatty scar involving the RV free wall

Cardiac Magnetic Resonance Imaging (MRI) showed enlarged RV with dyskinesia and reduced ejection fraction (RV ejection fraction 34.97%). An aneurysm close to the RV apex was also observed. Late-enhancement signal with Gadolinium showed diffused fatty infiltration in the RV free wall (Fig. 4). Left ventricular structure and function was normal (LV ejection fraction 62%).

**Fig. 5:**
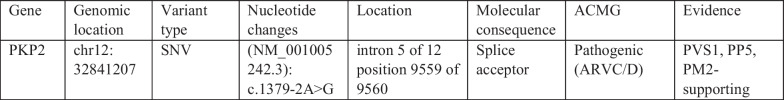
The results of molecular-genetic analysis

We then performed molecular-genetic analysis using next-generation sequencing (NGS) methods to identify gene mutations for further risk stratification. A mutation in the PKP2 gene which encodes Plakophilin-2 protein was identified (Fig. 5). The most prevalent cause of ARVC is a heterozygous mutation in this gene. So far, more than 200 mutations in the PKP2 gene have been documented, the majority of which are point mutations.

Finally, patient was diagnosed with ARVC according to the Modified Task Force criteria for ARVC proposed by the International Task Force of ESC and International Society and Federation of Cardiology in 2010. The specific diagnostic criteria were:

1. Major criterion: cardiac MRI found RV dyskinesia and RVEF 34.97%.
2. Major criterion: T wave inversion in leads V1–V3 on ECG
3. Minor criterion: 9899 PVCs on Holter ECG.
4. Major criterion: the PKP2 gene mutation.

After a thorough consideration of all risk factors as well as careful discussion with the patient’s parents, we decided to implant an implantable cardioverter defibrillator (ICD) for primary prevention of SCD. Patient was initially discharged on beta-blocker (bisoprolol 5 mg daily). After one month, amiodarone 100 mg daily was started when ICD check found many episodes of non-sustained VT.

## Follow-up and outcome

**Fig. 6:**
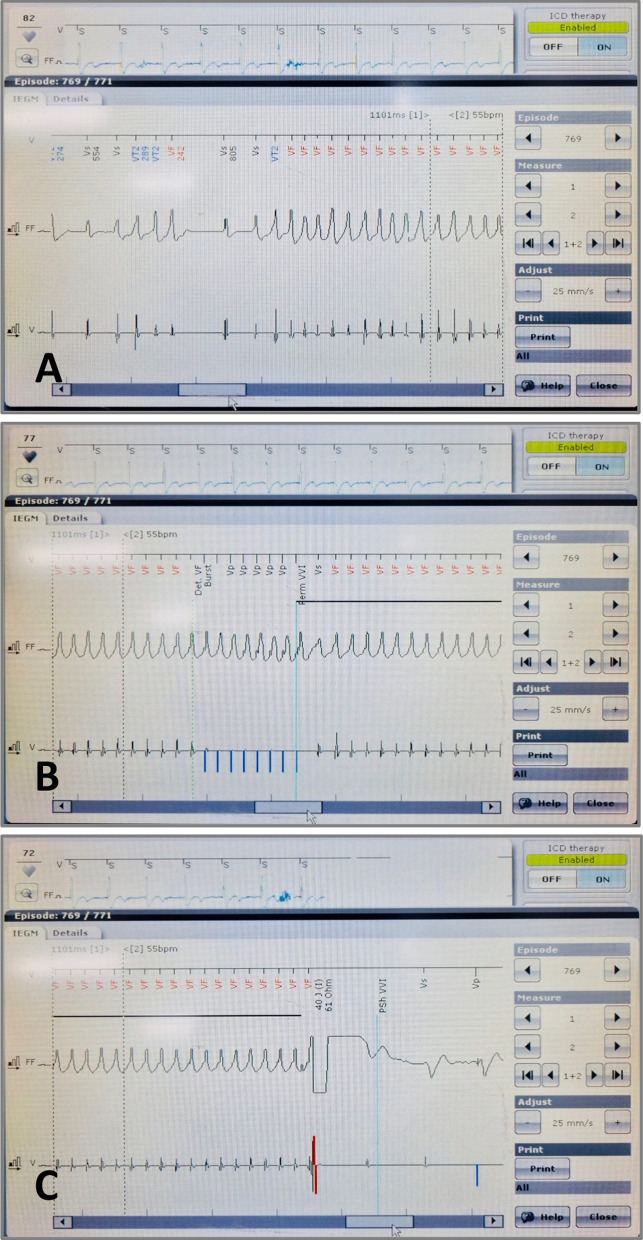
ICD detected (**A**) and delivered an appropriate shock (**C**) to terminate a fast VT or VF after a failed ATP (antitachycardia pacing) attempt (**B**)

At 6 months follow-up, the patient experienced a spontaneous episode of fast VT or VF, a potentially fatal ventricular arrhythmia, which was successfully terminated by an ICD shock (Fig. 6).

## Dicussion

ARVC is a heritable cardiomyopathy which may cause life-threatening ventricular arrhythmias leading to SCD. Fibrofatty replacement of RV myocardium remains to be the main histopathological characteristic of the disease [1, 2]. In 2010, the Task Force Criteria (TFC) for clinical diagnosis of the ARVC based on multiple factors was proposed by experts in the field of heart failure and cardiomyopathy. In 2019, HRS published an Expert Consensus Statement on Evaluation, Risk Stratification, and Management of Arrhythmogenic Cardiomyopathy, in which ACM is defined as the disease in heart muscle that involves the RV, left ventricle, or both [12]. The pathophysiological feature is fibrofatty infiltration of myocardium which may predispose patients to life-threatening arrhythmias and ventricular dysfunction. ACM are classified into 3 phenotypic variants including the classic ARVC, ALVC (arrhythmogenic left ventricular cardiomyopathy) and the disease involving both the ventricles. The diagnostic criteria for ARVC variant were based on major and minor criteria of the 2010 TFC [19]. Based on these diagnostic criteria, our patient met the diagnosis of definite ARVC.

In the 2010 TFC, gene mutation is considered as one of the major diagnostic criteria in the family history section. Specifically, identification of a pathogenic or likely pathogenic ACM mutation in the patient under evaluation is categorized as one of the major diagnostic criteria. Genetic analysis of our patient reveals the presence of a mutation of the PKP2 gene, which has been shown to be one of the mutations potentially related to ARVC.

The gene mutation in this patient changes a single nucleotide at the position NM_001005242.3: c.1379-2A > G belongs to the *PKP2* gene’s intron region. We used Clinvar and Varsome tool to characterize the mutation and applied the American College of Medical Genetics and Genomics (ACMG) criteria to determine its pathogenicity [14]. Based on these databases, this mutation belongs to a category of mutations that have the potential to cause ARVC. *PKP2* mutations are also the most common cause of ARVC in some populations. The *PKP2* gene encodes the synethis of a protein called plakophilin 2, which makes up structures called desmosomes. These structures form junctions that attach cells to one another. Abnormalities in the binding protein trigger myocardial remodeling and fat replacement process which may lead to arrthymia. *PKP2* mutations play an important role in the pathogenesis and progression of ARVC [15, 16].

According to the findings of Judith *et al.* the mutation detection prevalence in ARVC patients was 63%, with *PKP2* mutations accounted for 46% of all mutations [17]. In addition, a study on 90 ARVC patients in China by Jingru Bao *et al.* [18] revealed 57 subjects (63%) having genetic mutations, 58% of which occurred in the *PKP2* gene. Furthermore, the study’s findings demonstrated that ARVC patients with a gene mutation had a higher risk of VT than those without mutation. Similar finding was also observed in patients with and without *PKP2* gene mutation. This suggests that gene mutations, even if occurring in a single-gene, especially the *PKP2* gene, can be a predictor of the risk of cardiovascular events associated with ventricular arrhythmias and sudden death.

SCD due to ventricular arrhythmias can be prevented by ICD implantation [11]. Expert consensus has recommended that in ARVC patients, ICD is indicated for secondary prevention in patients with aborted cardiac arrest or hemodynamically unstable sustained VT, and for primary prevention in patients who have high risk of ventricular arrhythmias and SCD [12]. As our patient had no history of aborted SCD, ventricular arrhythmias associated syncope, sustained VT, or severely reduced LV ejection fraction on admission, risk factors for ventricular arrhythmias should be assessed to decide whether ICD implantation was necessary. According to the risk stratification guidelines recommended by the 2019 HRS Expert Consensus Statement on Evaluation, Risk Stratification, and Management of Arrhythmogenic Cardiomyopathy, our patient met only four minor risk factors for ventricular arrhythmias (male sex, > 1000 PVCs/24 h, RV dysfunction [34.97% on cardiac MRI] and proband status). Accordingly, the indication for primary prevention with an ICD for this patient was a class IIb recommendation with the level of evidence B. However, considering his family history with potential SCD in his older brother, the result of gene mutation and presuming that the disease’s severity may progress in the future, and with the family’s preference, a decision was made to implant an ICD for primary prevention of SCD. At 6 months post implantation, the patient experienced palpitations and near-syncope. A sustained fast VT or VF, a potentially fatal ventricular arrhythmia with an average cycle length of 229 ms, was detected and successfully terminated by an ICD shock. The decision of ICD implantation was finally proven to be appropriate for this patient.

## Conclusions

Gene mutations are known to be an important factor in the diagnosis of ARVC according to TFC 2010 criteria and later clinical guidelines. Although the role of gene mutation in risk stratification remains controversial, it still plays an important role in individualized risk stratification and management. In this case, gene mutation, along with other clinical factors and family history, was proven to be helpful in guiding treatment decisions of ICD implantation for primary prevention of SCD in a young patient diagnosed with ARVC.

## Data Availability

The data supporting this study is with the author and has been included within the manuscript.
